# Isolation of prostrate turfgrass mutants via screening of dwarf phenotype and characterization of a perennial ryegrass prostrate mutant

**DOI:** 10.1038/hortres.2016.3

**Published:** 2016-02-24

**Authors:** Junmei Chen, Chandra Thammina, Wei Li, Hao Yu, Huseyin Yer, Rania El-Tanbouly, Manon Marron, Lorenzo Katin-Grazzini, Yongqin Chen, John Inguagiato, Richard J. McAvoy, Karl Guillard, Xian Zhang, Yi Li

**Affiliations:** 1 College of Landscape Architecture and Arts, Northwest A&F University, Yangling 712100, China; 2 Department of Plant Science and Landscape Architecture, University of Connecticut, Storrs, CT 06269, USA; 3 College of Life Sciences, Hubei University, Wuhan 430062, China; 4 College of Horticulture, Northwest A&F university, Yangling 712100, China

## Abstract

Prostrate turf varieties are desirable because of their increased low mowing tolerance, heat resistance, traffic resistance and ground coverage compared with upright varieties. Mutation breeding may provide a powerful tool to create prostrate varieties, but there are no simple, straightforward methods to screen for such mutants. Elucidation of the molecular basis of the major ‘green revolution’ traits, dwarfism and semi-dwarfism, guided us to design a simple strategy for isolating dwarf mutants of perennial ryegrass (*Lolium perenne* L.). We have shown that gamma-ray-mediated dominant dwarf mutants can be easily screened for at the three-leaf stage. About 10% of dwarf mutant lines also displayed a prostrate phenotype at mature stages (>10 tillers). One prostrate line, Lowboy I, has been characterized in detail. Lowboy I had significantly shorter canopy, leaf blade and internode lengths compared with wild type. Lowboy I also exhibited greater tolerance to low mowing stress than wild type. Exogenous gibberellic acid (GA) restored Lowboy I to a wild-type phenotype, indicating that the dwarf and prostrate phenotypes were both due to GA deficiency. We further showed that phenotypes of Lowboy I were dominant and stably inherited through sexual reproduction. Prostrate turfgrass mutants are difficult to screen for because the phenotype is not observed at young seedling stages, therefore our method represents a simple strategy for easily isolating prostrate mutants. Furthermore, Lowboy I may provide an outstanding germplasm for breeding novel prostrate perennial ryegrass cultivars.

## Introduction

Perennial ryegrass (*Lolium perenne* L.) is an important turfgrass species, widely used for lawns and athletic fields because of its rapid establishment and attractive, leafy appearance.^1,2^ Because of its fast growth rate, perennial ryegrass requires frequent watering, fertilization and mowing, resulting in high establishment and maintenance costs.^3,4^ One strategy to reduce upkeep costs would be to use a dwarf variety of perennial ryegrass,^5–7^ however, none exists as of yet. Dwarf mutants can have lower upkeep costs due to their reduced vegetative growth, which necessitates less frequent mowing and fewer nutrients, and thus makes them less vulnerable to evaporation.

Another strategy would be to create a prostrate variety of perennial ryegrass. Prostrate turf has been shown to improve low mowing tolerance in bluegrass (*Poa* spp.).^8^ The horizontal growth of prostrate turf can protect leaf blades and sheaths, aiding in recovery from mowing stress. Young leaf blades, which are more photosynthetically active compared with older leaves, are more likely to be removed by mowing in upright varieties.^9^ Prostrate turf also appears to have higher heat tolerance because the plant crowns are shielded from the heat when they are far below the surface of the mat layer.^10^ Prostrate turf varieties have the potential to be more traffic resistant than upright varieties, whose leaves and stems are more likely to be damaged when subjected to traffic. Turf that grows in a prostrate manner appears to have greater ground coverage than upright turf because the flat-lying leaves can cover areas with lower turf density.

Induced mutation combined with selective breeding, a technique known as mutation breeding, is highly efficient for developing plant cultivars with improved traits. According to statistics released by the Food and Agriculture Organization of the United Nations in conjunction with the International Atomic Energy Agency, >2,600 novel cultivars of 214 plant species have been developed by mutation breeding.^11^ Gamma-ray irradiation was used to produce most of these mutant crop species.^12,13^ Gamma-ray irradiation has been used to induce changes in the morphology, cold tolerance and growth habits of centipedegrass (*Eremochloa ophiuroides*) and St Augustinegrass (*Stenotaphrum secundatum*).^14,15^ A variety of rhodes grass (*Chloris gayana* Kunth) created by gamma-ray irradiation exhibited a chlorophyll mutation in addition to sterility and other morphological variation.^16^ Dwarf, drought resistant and triploid Bermudagrass (*Cynodon dactylon*) mutants were also created using gamma-ray irradiation.^17–22^ Using mutation breeding to develop new cultivars of perennial ryegrass is difficult because it is self-incompatible.^23^ In order to screen for new traits, they must be the result of one or more dominant mutations. Recessive mutations will not manifest as visible phenotypes because mutant alleles will never exist in a homozygous state owing to the inability of perennial ryegrass to self-cross.

The ‘green revolution’ is a term applied to the period following the development of numerous dwarf, and semi-dwarf, varieties of important agricultural crop plants. It was later discovered that the semi-dwarf wheat and rice varieties used in the ‘green revolution’ were the result of dominant, or semi-dominant, mutations connected to the gibberellic acid (GA) signaling pathway.^24,25^ GA deficiency, or insensitivity can result in a dwarf phenotype, carrying along with secondary phenotypes associated with dwarfism. For example, the semi-dwarf wheat (*Triticum* spp.) and rice (*Oryza* spp.) varieties used in the ‘green revolution’ had a secondary phenotype of increased grain yield.

Here we report a simple strategy for isolating a prostrate mutant of perennial ryegrass through screening for dominant dwarf mutations. We also report a detailed characterization of one of the prostrate mutant lines that we recovered.

## Materials and methods

### Plant materials

‘Fiesta 4’ perennial ryegrass seeds were purchased from the Chas. C. Hart Seed Co. located in Wethersfield, CT, USA.

### Gamma-ray irradiation

Fiesta 4 perennial ryegrass seeds were immersed in tap water for 24 h and then irradiated with various doses of gamma rays (0, 2.5, 5.0, 7.5, 10.0, 15.0, and 20.0 kilorad (kr)) from a Cobalt-60 source in the Radiation Laboratory at the University of Massachusetts, Lowell, USA. Each treatment was performed in three replicates, with 1200 seeds per replicate, to determine the effects of radiation dosage on seed germination.

Irradiated seeds were pretreated at 4 °C for 2 days and allowed to germinate on moist paper towels at 25 °C under a 16-h light cycle (35–45 μmol m^−2^ s^−1^). The lethal dose of gamma-ray irradiation was determined based on seed germination rates after 21 days. The germination rate of each treatment was reported as an average of the three replicates.

Based on the calculated median lethal dose (LD_50_), a 9.0-kr dose was chosen for subsequent seed treatment. Ten kilograms of Fiesta 4 seeds were irradiated with 9.0 kr of gamma rays as described above. The irradiated seeds (M1 generation) were air-dried for 12 h and stored at 4 °C.

### Field planting and harvesting

M1 seeds were hand-broadcasted at a density of 1.5 kg per 100 m^2^ and grown to maturity at the University of Connecticut, Plant Science Research and Education Facility in Storrs, CT, USA. Progeny seeds were harvested, air-dried at room temperature and were stored at 4 °C.

### Identification of dwarf mutants

A total of 150 000 second-generation mutant seeds (M2) were treated with 289 μM GA_3_ for 10 h to promote uniform germination. Seeds were then rinsed with tap water and held at 4 °C for 14 days. Cold-treated seeds were germinated and grown in shallow black germination trays (56×29×3.3 cm) containing Promix potting soil (Premier Horticulture Inc., PA, USA) in the greenhouse, with 3,000 seeds per tray. Two to three weeks later, when seedlings reached the three-leaf stage, dwarf mutants were easily identified. The seedlings that had their leaf blades at least 30% shorter than the estimated average were transferred to 7.62 cm plugs and were allowed to grow further for evaluation. Plants that maintained a dwarf phenotype into maturity, as well as in their vegetatively propagated progeny, were named and used for detailed characterization.

### Morphological characterization of Lowboy I M2 plants under field conditions

One of the dwarf mutants, Lowboy I, was selected for further characterization. M2 Lowboy I and wild-type Fiesta 4 plants were vegetatively propagated and grown in the greenhouse for 2 months in 7.62 cm plug trays. Plugs with 8–10 tillers each were planted in the field in September 2011, 2012 and 2013. Plugs were planted in rows and spaced 30 cm apart, with no additional fertilization or irrigation after establishment. Field test plots used a randomized design with six replicates per mutant line and wild type.

At the end of each June, from 2012 to 2014, turf planted in the September of previous year was carefully dug out and soil was washed from the roots. Ten representative tillers were picked from each of the six replicates. To measure leaf blade length, leaf blade width and internode length, the tallest three leaves and internodes from each of the 10 tillers were measured. Mean leaf sizes and internode lengths were calculated for each replicate. Canopy lengths, root lengths and ratios of root/shoot biomass were determined using six replicates. For the root/shoot biomass, plant materials were oven-dried at 70 °C for 10 days and were then weighed. Data were reported as the mean of all replicates.

### Evaluation of tolerance to low mowing height

In September 2011, Lowboy I M2 and wild-type plants were vegetatively propagated on Woodbridge fine sandy loam soil, in a 28×56 cm area at a density of 16 tillers per 100 cm^2^ in the greenhouse. In May 2012, greenhouse-grown 8-month-old Lowboy I M2 and wild-type sod strips (28×56 cm) were randomly planted on Woodbridge fine sandy loam soil under field conditions. Each test line was planted in triplicate. A distance of 30 cm was left between sod strips in a row and between rows. Sod strips were watered as needed until fully established. Upon establishment, mowing heights for Lowboy I M2 and wild-type sod strips were gradually lowered from 7.62 to 1.90 cm. From June until August (2012–2015), test plots were mowed twice per week with a John Deere JS60 rotary push mower. Turf density, leaf texture and turf quality were determined in the final week of July 2013 according to the procedures described by Morris.^26^

### Morphological evaluation of Lowboy I M3 progeny plants

Lowboy I M2, Gamma-2 M2 and wild-type plants were vegetatively propagated in the greenhouse for the controlled crossings Lowboy I M2 (♀)×wild-type (♂) and Lowboy I M2 (♀)×Gamma-2 M2 (♂). Gamma-2 is another dwarf mutant isolated during the same initial screening as Lowboy I. Plugs of size 7.62 cm (8–10 tillers each) were planted in the field during September 2012. A distance of 46 cm was left between each row and 18 cm was left between plants within a row. Plastic was used to wrap cages surrounding parental plants in order to prevent undesired cross-pollination, after which plants were manually agitated with one another to facilitate pollination. Seeds were harvested separately from each plant, air-dried at room temperature and stored at 4 °C.

Seeds from each crossing were germinated in Pen packs (15×11×5 cm; K&C Plastics, Leominster, MA, USA) containing Promix potting soil. After 60 days, the progeny plants were sorted based on height. M3 progeny were divided into two groups: tall and short. Six individual plants were selected from each of the following groups: wild type, Lowboy I M2, Gamma-2 M2, tall progeny of Lowboy I M2 (♀)×wild type (♂), short progeny of Lowboy I M2 (♀)×wild type (♂) and progeny of Lowboy I M2 (♀)×Gamma-2 M2 (♂). These plants were vegetatively propagated in 7.56-cm plug trays containing Promix potting soil. After 60 days, measurements of shoot length, root length, tiller number and dry root/shoot biomass were taken.

### GA treatment

M2 Lowboy I and wild-type Fiesta 4 plants were vegetatively propagated and grown in the greenhouse for 2 months in 7.62-cm plug trays. Plants were then cut to a height of 5 cm, after which they were sprayed once a week with 10 ml (per plant) of a 5 mg/l GA_3_ solution. The experiment was done with six replicates for the mutant line and wild-type plants. Measurements and photos were taken after 3 weeks of GA_3_ treatment.

### Statistical analysis

Analysis of variance was performed on data collected from greenhouse-grown and field-grown plants using IBM SPSS 19.0 (IBM Corp., Somers, NY, USA). When sufficient differences (*P*<0.05) were observed, Fisher’s least significant difference test (*P*=0.05) was performed to detect differences between treatments.^27^ Comparison between the mean turf quality characteristics in low mowing experiments of Lowboy I M2 and wild-type plants used two-tailed Student’s *t*-tests.

## Results

In an initial experiment, a range of gamma-ray doses (0–20 kr) was used to treat Fiesta 4 perennial ryegrass seeds, after which the germination rate was tested. Germination rate data were collected 21 days post germination. There was a strong negative correlation between seed germination rate and gamma-ray dosage (Table 1). Compared with untreated seeds, germination rates of 7.5- and 10.0-kr irradiated seeds were reduced by 39.96% and 58.68%, respectively (Table 1). A gamma ray dose resulting in a 50% reduction in seed germination (LD_50_) is often used for mutagenesis experiments.^28,29^ We estimated that a 9.0-kr dose would reduce germination rates by ~50%, and was therefore chosen for subsequent seed treatment. First-generation mutant (M1) seeds were planted in the field and grown to a flowering stage, after which they were allowed to cross randomly. The resultant progeny seeds (M2) were harvested and germinated.

**Figure 1:**
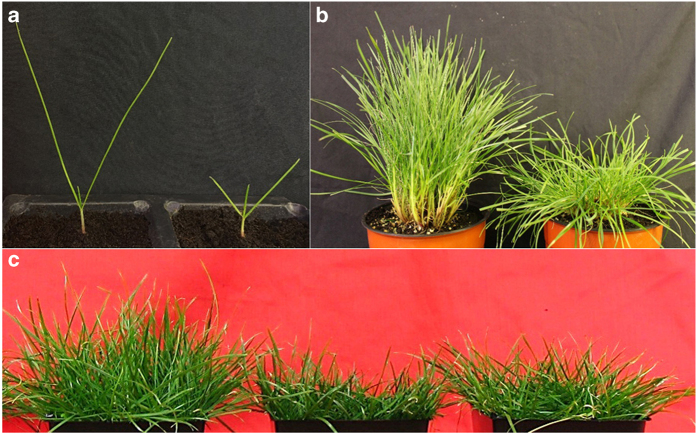
Comparisons of Lowboy I M2, Lowboy I M3 and wild-type (WT) Fiesta 4 perennial ryegrass under various experimental conditions. (**a**) An 8-week-old Lowboy I M2 mutant (right) exhibited dwarf characteristics compared with WT (left). (**b**) Lowboy I plants (right) had dwarf and prostrate phenotypes compared with WT (left). (**c**) Lowboy I M3 plants retained short growth and prostrate characteristics (from left to right: WT, Lowboy I M2 and Lowboy I M3).

After germination, M2 seeds were visually screened for a dwarf phenotype (>30% reduction in leaf blade length) at the three-leaf stage of seedling development (Figure 1a). More than 75 dwarf mutant lines were recovered from 150 000 M2 seedlings, with 51 lines maintaining their dwarfism both into maturity and after vegetative propagation. At the vegetative maturity stage (>10 tillers), ~10% of dwarf mutants displayed a prostrate secondary phenotype (data not shown). One of these mutant lines, named Lowboy I (Figure 1b), was further characterized.

Lowboy I plants displayed a consistent growth habit in the field over a 3-year evaluation period, from 2012 to 2014. Lowboy I developed canopy lengths that were only 65–69% of wild type (Table 2). Similarly, Lowboy I had leaf blade lengths, leaf blade widths and internode lengths reduced to 40–45%, 16–18% and 51–56% of wild type, respectively. Root lengths were not significantly different between Lowboy I and wild-type, and the ratios of dry root/shoot biomass were slightly higher for Lowboy I.

In field tests of tolerance to low mowing height (1.9 cm), Lowboy I displayed significantly higher turf density, better leaf texture, and better turf quality than wild type in 2012, 2013 and 2014 (data from 2013 are mentioned in Table 3). Turf density, leaf texture and turf quality were determined according to the procedures described by Morris.^26^ The Lowboy I had a high turf density, whereas wild-type controls had only medium turf density. Lowboy I had close to the best possible turf quality, whereas wild type had quality slightly above the acceptable level. The fact that Lowboy I leaves were narrower than those of wild type improved the appearance of Lowboy I under low mowing height conditions (Table 3). Lowboy I plants also had shorter internodes lengths than wild-type plants, with more leaves retained on the stems at low mowing height.

Wild-type Fiesta 4 perennial ryegrass (♂) was crossed with Lowboy I M2 ryegrass (♀) to produce M3 progeny in order to determine whether the phenotype observed in Lowboy I M2 plants was stably inherited. We characterized the M3 progeny under greenhouse conditions and observed that ~50% of the M3 plants from the crosses between the wild type (♂) and Lowboy I (♀) were similar to the wild-type controls; these were categorized as tall progeny. The other 50% were shorter than wild-type plants and were categorized as short progeny. Although the leaf blade lengths of the short group were significantly shorter than those of wild type, they were still significantly longer than those of Lowboy I M2. The tiller numbers of tall and short progeny were not significantly different from those of Lowboy I M2 and wild-type plants (Figure 1c and Table 4). The root lengths of tall progeny were not significantly different from those of Lowboy I M2 and wild-type plants, however, short progeny had significantly shorter root lengths than Lowboy I M2 plants. The dry root/shoot biomasses of short progeny were similar to those of Lowboy I M2 plants and were significantly greater than those of wild type. We also crossed Lowboy I (♀) with Gamma-2 (♂), another dwarf mutant, and were able to get progeny displaying a more compact dwarf phenotype than found in either parental line (Table 4).

**Figure 2:**
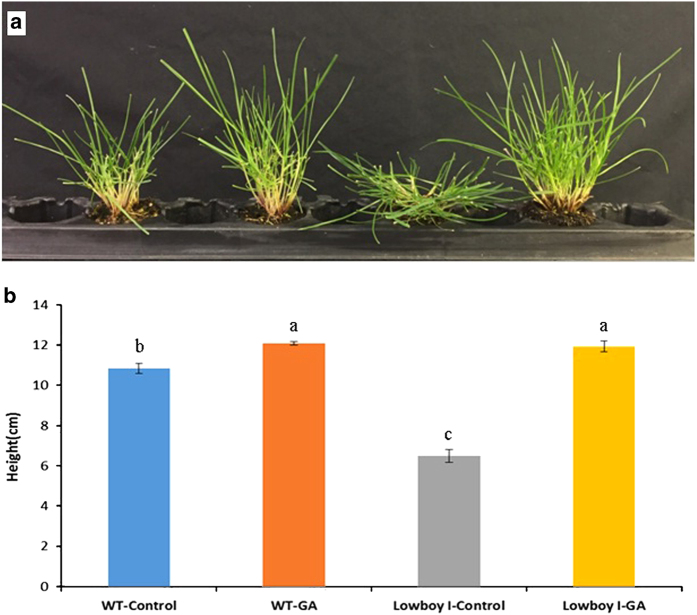
GA_3_ treatment of Lowboy I M2 and wild–type (WT) Fiesta 4 perennial ryegrass. (**a**) GA_3_ treatment was able to restore Lowboy I mutant plants to a WT phenotype (from left to right: untreated WT, WT treated with GA_3_, untreated Lowboy I and Lowboy I treated with GA_3_). (**b**) Lowboy I mutants treated with GA_3_ became as tall as WT treated with GA_3_. Each bar represents the mean of six replicates grown under greenhouse conditions, each replicate was one representative plant. Data were collected after 3 weeks of weekly GA_3_ treatment. Bars with the same letter above them are not significantly different from each other according to Fisher’s least significant difference (*P*=0.05).

In order to verify that the phenotype of Lowboy I was a result of GA deficiency, Lowboy I M2 plants were treated with GA_3_, and their subsequent growth and morphology was compared with those of wild-type controls. It was found that, over a 3-week period, GA_3_-treated Lowboy I plants were restored to the same height as wild-type plants. In addition, the prostrate phenotype observed in Lowboy I was completely abolished following GA_3_ treatment (Figures 2a and b). These results demonstrate that GA_3_ treatment is sufficient to restore Lowboy I to a wild-type phenotype, indicating that the mutation found in Lowboy I results in GA deficiency.

## Discussion

Prostrate turf has advantages over upright turf in a number of applications, most notably for low mowing tolerance and improved heat resistance. Prostrate turf can also be more traffic resistant, as leaf blades and stems are more likely to be pressed flat than damaged when in high traffic environments. Prostrate turf can also have better ground coverage, as leaves that lie flat upon the ground obscure areas where turf density is lacking. Prostrate phenotypes can be difficult to screen for in mutation breeding programs due to their delayed presentation. However, as shown in this study, ~10% of dwarf mutants display a prostrate phenotype at mature stages (>10 tillers). Therefore, it is possible to initially screen for dwarfism, which can be seen at the three-leaf stage, in order to isolate prostrate mutants.

Mowing accounts for 60% of the overall cost of lawn maintenance.^3,30^ Various methods have been used to reduce mowing costs, including less frequent mowing. However, there are often functional or aesthetic reasons as to why less frequent mowing is undesirable. The most effective way to reduce costs while maintaining a presentable appearance is to use a dwarf turf variety. The Lowboy I perennial ryegrass mutant, reported here, has the potential to reduce mowing frequency significantly. Based on the growth rate of Lowboy I, we expect that its mowing frequency may be reduced by half or two thirds compared with wild type. Our mowing experiments demonstrated that Lowboy I displayed significantly improved tolerance to low mowing height when compared with wild type, with improved turf density, leaf texture and turf quality in a 3-year field study. Lowboy I plants had shorter internodes than wild-type controls, with more leaves retained on the stems after mowing to 1.9 cm. The narrower leaves of Lowboy I also resulted in a better visual appearance after low mowing. Although perennial ryegrass is relatively resistant to low mowing and has been used on golf course fairways, improvement in turf quality at mowing heights under 1.9 cm would make a perennial ryegrass mutant such as Lowboy I a stellar choice for athletic fields as well as golf teeing grounds and fairways.

Perennial ryegrass prefers relatively high amounts of water and fertilizer compared with other turf varieties.^1,31^ Lowboy I displayed dwarfism while maintaining a strong root system under both greenhouse and field conditions. The characteristics of this phenotype that are consistent with those of other dwarf turf varieties have been shown to require less irrigation and fertilization.^17–19,32^ However, additional experiments are needed to determine whether Lowboy I can tolerate reduced water and fertilizer.

The preferred method for growing perennial ryegrass is through seed dispersal. Trait inheritance through sexual reproduction is a basic requirement for commercially relevant cultivars.^3,33^ We have demonstrated that the dwarf phenotype observed in Lowboy I is a dominant trait and can be stably inherited by progeny through sexual reproduction. Furthermore, Lowboy I and Gamma-2 hybridize to produce seeds with a severe dwarf phenotype. It is important to note that Lowboy I plants produced an equivalent seed yield to wild type. These results suggest that Lowboy I can be used as a parental line to cross with other dwarf lines in order to produce progeny with enhanced dwarf phenotypes.

The strategy detailed in this paper for identifying a dwarf, prostrate phenotype in the M2 generation of perennial ryegrass is dependent upon a dominant mutation as the source of the phenotype. Phenotypes resulting from recessive mutations are invisible to screening because perennial ryegrass is self-incompatible. For a recessive mutation to become visible, it must exist in a homozygous state. Without the ability to self-cross, mutant alleles cannot become homozygous in perennial ryegrass.

Through the GA treatment of mutant plants, we were able to confirm that Lowboy I mutants were GA deficient, as they were restored to a wild-type phenotype following the application of GA_3_ to the leaves. GA deficiency was found to be responsible for both the dwarf and prostrate phenotypes observed in the Lowboy I mutant. This is the first reported case of a prostrate phenotype being linked to GA deficiency in perennial ryegrass.

Previous studies in *Arabidopsis*, maize, rice and other plant species have shown that loss-of-function mutations in GA biosynthetic genes reduce stem and leaf length growth.^34–36^ It has been reported that in barley (*Hordeum vulgare*), the loss of function of *GA3ox1* gene, the enzyme responsible for the last step of GA biosynthesis, led to both dwarfism and prostrate growth.^37^ Ordonio *et al.*^38^ reported that in their M2 sorghum mutant population, they isolated several prostrate dwarf mutants. Their characterization showed that GA deficiency was the root cause behind both phenotypes. The GA deficiency of these mutants was due to a loss-of-function mutation in one of four genes (*SbCPS1*, *SbKS1*, *SbKO1* and *SbKAO1*) that are involved in the early steps of GA biosynthesis. They used GA treatments to restore the mutant plants to a wild-type phenotype. All of these mutant studies, in addition to the analysis of Lowboy I reported here, demonstrate that prostrate growth is one of the pleiotropic effects of GA deficiency in some monocot plant species.

However, the mutations in barley, wheat, and sorghum were all recessive, whereas the mutation in Lowboy I is likely dominant. Unlike the sorghum prostrate mutants, it is more likely that the mutant gene found in Lowboy I is a negative regulator of the GA biosynthetic pathway. Cloning and characterization of the mutant gene should provide insight into the molecular basis for dwarfism and prostrate growth in Lowboy I.

## Conclusions

Gamma-ray irradiation has been used to create dominant mutations for dwarfism in Fiesta 4 perennial ryegrass. One mutant, Lowboy I, displayed both dwarfism and prostration, with improved turf density, turf quality and resistance to low mowing height. The prostrate and dwarf phenotypes of Lowboy I were completely eliminated with exogenous GA_3_ application, demonstrating that both traits are most likely the result of GA deficiency. The observed Lowboy I phenotypes are also stably inherited in progeny through sexual reproduction. In this study, we demonstrate that mutation breeding can be used to create dominant or semi-dominant mutants in turfgrasses. Furthermore, we show that screening for prostrate mutants of turfgrass can be simplified with an initial screen for dwarfism.

## Figures and Tables

**Table 1:** Effect of gamma-ray dosage on the germination of Fiesta 4 perennial ryegrass seeds

*Dosage (kr)*	*Germination rate (% ±s.e.)*
0	92.39±2.02 (a)
2.5	78.93±0.71 (b)
5.0	70.03±1.52 (c)
7.5	55.47±1.61 (d)
10.0	38.17±1.03 (e)
15.0	24.37±1.45 (f)
20.0	19.10±0.15 (g)

Gamma radiation of perennial ryegrass seeds led to differing germination rates. Each value represents the mean germination rates of three replicates. Values in the same column followed by the same letter are not significantly different from each other according to Fisher’s least significant difference (*P*=0.05).

**Table 2:** Characterization of morphological features of Lowboy I M2 and wild-type perennial ryegrass grown in the field during 2012 and 2013

*Genotype*	*Canopy length (cm, mean±s.e.)*	*Root length (cm, mean±s.e.)*	*Leaf blade length (cm, mean±s.e.)*	*Leaf blade width (cm, mean±s.e.)*	*Internode length (cm, mean±s.e.)*	*Dry root: shoot biomass ratio (mean±s.e.)*
*Year 2012*						
Wild type	68.33±0.33 (a)	33.00±0.58 (ab)	13.07±0.47 (a)	0.32±0.02 (a)	7.71±1.14 (a)	0.118±0.016 (b)
Lowboy I	44.33±1.86 (b)	36.00±0.58 (a)	7.16±0.40 (b)	0.27±0.01 (b)	3.79±0.79 (b)	0.134±0.011 (b)
						
*Year 2013*						
Wild type	67.00±1.15 (a)	32.33±1.20 (b)	12.99±0.06 (a)	0.33±0.02 (a)	8.06±0.07 (a)	0.126±0.012 (b)
Lowboy I	46.00±0.58 (b)	29.67±1.45 (b)	7.78±0.12 (b)	0.27±0.01 (b)	3.58±0.06 (b)	0.162±0.015 (a)

Data were collected in June 2012 and June 2013 (at plant maturity). Each value represents the mean of six replicates, comprised of one plant each. Values in the same column followed by the same letter are not significantly different from each other according to Fisher’s least significant difference (*P*=0.05).

**Table 3:** Comparison of turf quality characteristics of Lowboy I M2 and wild-type perennial ryegrass after low mowing tolerance testing in 2013

*Genotype*	*Turf density (mean±s.e.)*	*Leaf texture (mean±s.e.)*	*Turf quality (mean±s.e.)*
Wild type	5.40±0.31	5.01±0.14	6.17±0.17
Lowboy I	7.20±0.41*	6.90±0.16*	8.00±0.58*

Each value represents the mean of three replicates. Turf density is a measure of the number of aerial shoots per unit area. Turf density rating: 1=less dense; 9=more dense. Leaf texture is a measure of the width of leaf blades. Fine textured turf grasses have narrow leaves. Leaf texture rating: 1=course texture; 9=fine texture. Turf quality is a composite score determined by the collective contribution of shoot density and leaf texture, smoothness and color. Turf quality rating: 1=poorest possible turf quality; 9=best possible turf quality, 5=minimum acceptable value for turf quality. Asterisks represent a significant difference from the wild-type samples according to Student’s *t*-test (*P*⩽0.05).

**Table 4:** Morphological characteristics of M3 progeny of Lowboy I M2 (♀)×wild-type (♂) and Lowboy I M2 (♀)×Gamma-2 M2 (♂) under greenhouse conditions

*Genotype*	*Leaf blade length (cm, mean±s.e.)*	*Root length (cm, mean±s.e.)*	*Tiller number (mean±s.e.)*	*Ratio of root/shoot dry mass (mean±s.e.)*
Wild type	13.46±0.40 (a)	14.50±0.29 (ab)	12.33±0.33 (a)	0.283±0.003 (b)
Lowboy I M2	6.53±0.12 (d)	14.72±0.15 (a)	12.67±0.33 (a)	0.396±0.006 (a)
Lowboy I M2×wild type (tall group)	12.3±0.80 (a)	14.10±0.12 (ab)	13.00±0.58 (a)	0.263±0.034 (b)
Lowboy I M2×wild type (short group)	8.04±0.02 (b)	13.67±0.27 (b)	12.00±0.00 (a)	0.403±0.003 (a)
Gamma-2 M2	7.02±0.02 (c)	14.17±0.38 (ab)	13.00±0.58 (a)	0.400±0.002 (a)
Lowboy I×Gamma-2	5.96±0.05 (e)	13.67±0.33 (b)	12.67±0.33 (a)	0.356±0.015 (a)

Each value represents the mean of six replicates grown under greenhouse conditions, each replicate was one representative plant. Data were collected after 4 months of growth. Values in the same column followed by the same letter are not significantly different from each other according to Fisher’s least significant difference (*P*=0.05).
